# The relationship between intraoperative hypothermia and the ccurrence of surgical site infections: a meta-analysis of observational studies

**DOI:** 10.3389/fsurg.2024.1436366

**Published:** 2024-07-11

**Authors:** Jing Ju, Feng Zhou, Zhenzhi Wang

**Affiliations:** ^1^ Operation Room, Rainbow Hospital of Xianyang, Xianyang, Shaanxi Province, China; ^2^ The Affiliated Hospital of Shaanxi University of Chinese Medicine, Xianyang, Shaanxi Province, China; ^3^ The Acupuncture and Tuina School, Chengdu University of Traditional Chinese Medicine, Chengdu, China

**Keywords:** preoperative hypothermia, surgical site infection (SSI), systematic review, meta-analysis, risk factor

## Abstract

**Objective:**

Inconsistent evidence exists regarding the association between intraoperative hypothermia and incidence of surgical site infection (SSI). This study aimed to determine the association between intraoperative hypothermia and SSI.

**Materials and methods:**

A systematic review was conducted using Embase, PubMed, and Web of Science to identify observational studies evaluating the risk of SSI in patients with intraoperative hypothermia. The primary outcome measure was the diagnosis of SSI within 30 days of surgery. The pooled risk ratio was estimated using a fixed- or random-effect meta-analysis. Sensitivity analyses were performed to examine the impact of the structural design of preoperative warming on the pooled risk of SSI.

**Results:**

Five studies representing 6,002 patients were included in the present meta-analysis. Intraoperative hypothermia was not associated with SSI risk in patients (HR = 1.22, 95% CI: 0.95–2.24, *P *= 0.119). The pooled hazard ratio showed that intraoperative hypothermia did not result in a higher risk of SSI.

**Conclusions:**

Intraoperative hypothermia was not associated with the risk of SSI. Further studies using objective exposure measurements are required to confirm these results.

## Introduction

Intraoperative hypothermia, generally defined as a core body temperature <36  °C during surgery, can occur at any stage during the perioperative period. This is a common, but preventable complication of surgery (1, 2). During surgical anesthesia, hypothermia is mainly caused by anesthesia-induced changes in thermoregulatory physiology, decreased metabolism, infusion of fluids or blood products, and patient exposure to a cold operating room. It has been found that hypothermia has deleterious effects on different physiological systems and functions of the patient (3, 4). The complications of perioperative hypothermia can affect various organs and systems and may lead to increased morbidity and mortality, increased healthcare costs, and reduced patient satisfaction with hypothermia (5).

Surgical site infection (SSI) is responsible for significant financial burden and ineffectiveness in patients. SSI is defined as an infection occurring at the surgical site within 30 days of surgery (6). An SSI is one of the adverse events associated with hypothermia (7, 8). The development of SSI has substantially increased the clinical and economic burden of surgery. The financial burden of surgery is increasing due to the direct costs incurred by prolonged hospitalization of the patient, diagnostic tests, and treatment. Certain patients may also require reoperation after the development of SSI, which is associated with considerable additional costs (9). Broex et al. demonstrated that in European hospitals, patients who develop an SSI constitute approximately double the financial burden of patients who do not develop an SSI (10). In addition, they reported that the length of hospitalization was more than twice as long for patients with SSI than for uninfected patients. SSIs represent an opportunity cost to hospitals by displacing hospital resources that would otherwise be spent elsewhere and cause a delay of subsequent surgeries. Following their discharge from the hospital, patients with SSIs may also rely on healthcare from other community care services, which further contributes to the economic burden of infection.

SSIs have been consistently reported as one of the most common healthcare-acquired infections, with up to 60% of infections being preventable (11, 12). As a result of these findings, extensive efforts have been made by hospitals and surgical quality managers worldwide to maintain the average body temperature in patients undergoing surgery of all ages. Although some studies have confirmed a clear association between perioperative hypothermia and subsequent SSI in adults (13–15), others have suggested no correlation between intraoperative hypothermia and SSI (16, 17). In this study, we aimed to determine the association between intraoperative hypothermia and the risk of SSI, quantify the effect of perioperative hypothermia on the occurrence of SSI, and compare the evidence from observational studies.

## Methods

Our reporting items followed the Preferred Reporting Items for Systematic Reviews and Meta-Analyses (PRISMA 2020) guidelines (18). The study protocol was not registered.

### Search strategies

The PubMed, Embase, and Web of Science databases were searched using a computer with subject terms or in combination with accessible terms. The search period was from the time of database creation to October 1, 2023, and the English search terms included: “Surgical Wound Infection,” “Infections, Surgical Wound,” “Infection, Postoperative Wound,” “Surgical Site Infections,” “Hypothermia,” “Hypothermia, Accidental,” and “Accidental Hypothermias” without filters or language restrictions. The reference lists of the included articles and relevant reviews were reviewed.

### Eligibility criteria

Reports meeting several specific criteria were considered for inclusion: (a) the following adverse events were reported regardless of surgery in the study population: SSI; (b) inclusion of at least one low-temperature group and one standard group, and (c) low temperature is defined as below 36 °C, and (d) all clinical studies were eligible, including case reports and cohorts, case-controls, one-arm, prospective, and retrospective studies.

Reports that met other criteria were excluded: (a) studies without original data, including reviews, meta-analyses, clinical study protocols, reviews, or editorials; (b) research involving non-human research subjects and molecular, cellular, or animal experiments; (c) inappropriate grouping (e.g., grouping by using/not using warming methods, but inadequate reporting of actual occurrence/absence of intraoperative hypothermia), (d) incomplete data (e.g., lack of OR/HR), (e) induction of hypothermia for therapeutic purposes (e.g., unexpected brain injury, myocardial disease), and (f) repeated publication.

### Data extraction

Two researchers independently read all included studies for information screening, retrieval, and quality assessment. Disagreements were resolved through discussion or introduction by a third author. The following entries were retrieved: author name, year of publication, country, population, study design, sample size, intraoperative nadir (i.e., minimum) temperature, incidence of hypothermia, incidence and primary outcome of SSI, time of surgery, and development of surgery.

### Quality assessment and risk of bias

For cohort and case-control designs, the quality of the included studies was assessed using the Newcastle–Ottawa Scale (NOS), which includes eight specific quality items in three domains: selection, comparability, and exposure/outcome (19) (Table 1). All items were given one star except for comparability, which was given a maximum of two stars. A score of ≥7 was considered as high quality. Otherwise, it was considered to be of low quality. A median/mean follow-up period of ≥5 years or a maximum follow-up period of ≥10 years was considered adequate. Begg's and Egger's tests were used to assess the publication bias. There was no publication bias when *P* values for both were ≥0.05. The trim-and-fill method was used to evaluate the impact of potentially unpublished studies.

**Table 1:** Quality assessment of included studies.

Study (cohort)	Representativeness of exposed cohort	Selection of non-exposed cohort	Ascertainment of exposure	Outcome not present before study	comparability	Assessment of outcome	Follow-up long enough	Adequacy of follow up	Quality score
Seamon et al. (13)	^a^	^a^	^a^	^a^	^b^	^a^			7
Frisch et al. (14)	^a^	^a^	^a^	^a^	^b^	^a^	^a^		8
Eng et al. (15)	^a^	^a^	^a^	^a^	^b^	^a^			7
Baucom et al. (17)	^a^	^a^	^a^	^a^	^b^	^a^			7
Study (case-control)	Case definition	Representativeness of the cases	Selection of controls	Definition of controls	Comparability	Ascertainment of exposure	Same method	Non-response rate	Quality score
Brown et al. (16)	^a^	^a^	^a^	^a^	^b^	^a^	^a^	^a^	9

Follow-up long enough: ^a^Median/mean follow-up of more than 5 years or maximum follow-up of more than 10 years was considered enough.

Adequacy of follow up: ^b^A follow-up rate of >80% and a descriptive analysis of those who were missed was considered adequate.

### Statistical methods

For each meta-analysis, we estimated the summary effect size and 95% CI using fixed- and random-effects models (20, 21). We also assessed the 95% prediction interval, which further accounted for between-study heterogeneity, and evaluated the uncertainty of the effect expected in a new study addressing the same association (22, 23). To assess the effects reported in the studies, a summary of effect size was calculated. Various effect sizes were encountered, including OR and HR. HR was chosen as the effect size for analysis due to its common use in long-term follow-up studies to assess event risk. HR is more suitable for capturing the impact of time on event occurrence compared to OR. Therefore, HR was selected for statistical analysis and pooling to conduct a comprehensive assessment of the overall effect in the included studies. The data were analyzed using STATA 16.0 software; the effect indicators were expressed as HR, and interval estimates were described as 95% CI. The I^2^ test was chosen for heterogeneity, and if there was no heterogeneity in the data (I^2^ ≤ 50% and *P* ≥ 0.1), a fixed-effects model was selected for the analysis; conversely, a random-effects model was used. The results of the meta-analysis were expressed as forest plots. A *P* value < 0.05 was considered as statistically significant. A subgroup analysis was performed to determine the cause of clinical heterogeneity. Due to the variety of interventions, study designs, and other factors involved in the included studies, a random-effects model was used to assess the combined effect sizes of the efficacy variables. The impact of individual studies on the overall results was evaluated using a sensitivity analysis (paper-by-paper exclusion) or descriptive analysis. Begg's and Egger's tests were used to assess the publication bias. If missing or incomplete data were found, the researchers of the original study were contacted.

### Publication bias

Funnel plots were not constructed because the number of included studies was less than ten. The *P*-values for Begg's and Egger's tests were 0.086 and 0.168, respectively, indicating no potential publication bias for the current study.

## Results

### Literature search

**Figure 1:**
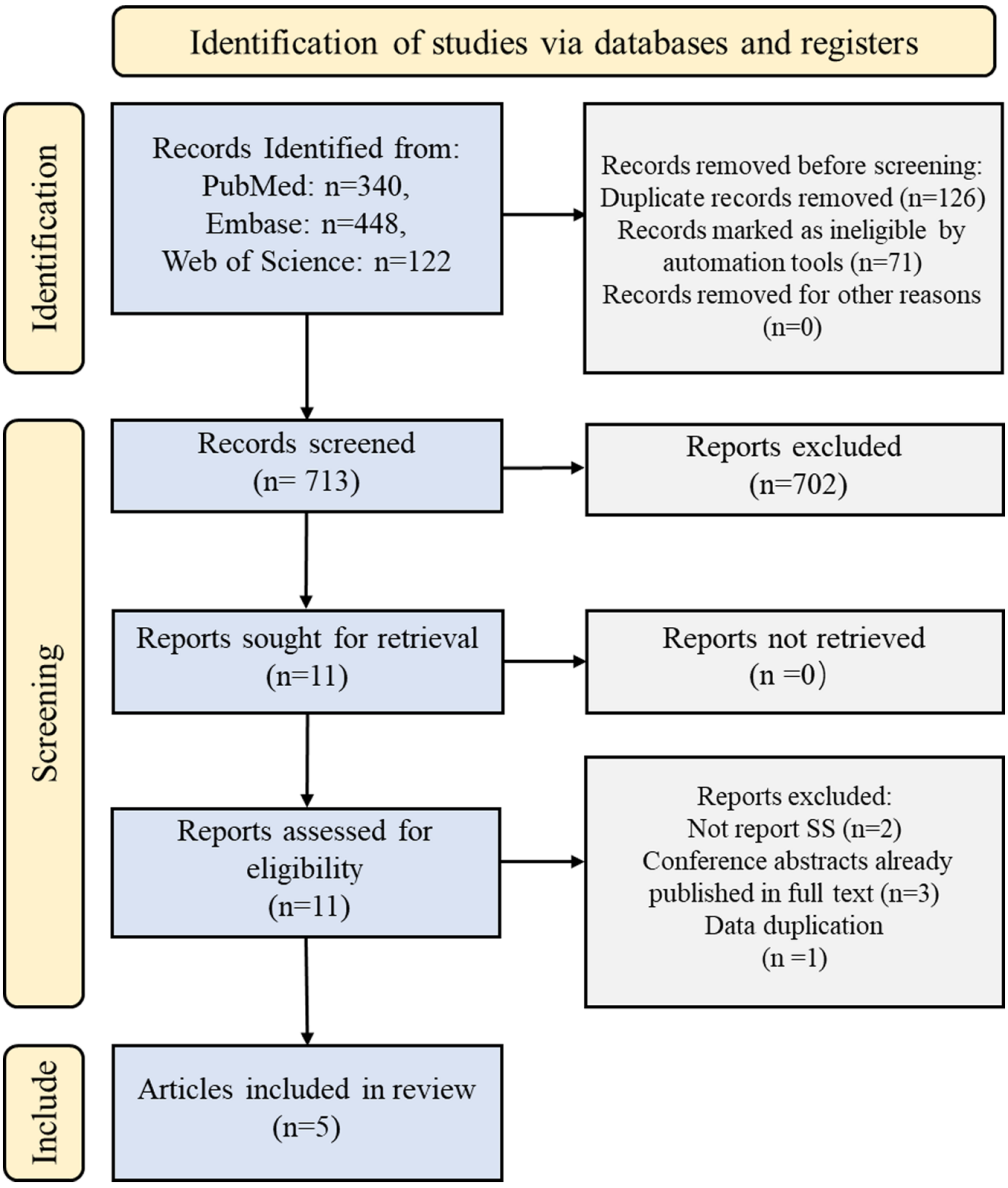
Flowchart of study selection.

In total, 910 records were identified using the initial search strategy. After excluding duplicates and reviewing titles, abstracts, and full text, five relevant studies were selected according to the inclusion and exclusion criteria (13–17). The detailed search process is illustrated in Figure 1.

### Basic characteristics

In total, 910 records were screened. Of these, 702 studies were excluded on the basis of their abstracts. Eleven full-text articles were assessed and five studies were included in the meta-analysis. The baseline characteristics of the included studies are summarized in Table 2. All the studies were performed in the USA. Four studies were cohort studies and one was a case-control study. Intraoperative hypothermia was <36° in four studies and <35° in one study. The mean age at baseline varied from study to study, ranging from 30 to 77 years. Only one study reported the duration of follow-up. The remaining four studies did not mention the duration of follow-up.

**Table 2:** General study characteristics of the included studies.

Study, year	Design	Country	Sources of participants	No. (M/F)	Numbers and percentages of SSI	Mean/median age years	Intraoperative hypothermia description	Reference category definition	Mean/median intraoperative temperature in SSI/NO SSI	Cutoff time of exposure	Follow up (CP/control(weeks)	HR	95%LL	95%UL
Seamon et al. (13)	Cohort	America	All trauma patients who underwent urgent laparotomy during July 2003 until July 2008 at T emple University Hospital	524 (NP/NP)	189, 36%	30.9 ± 12.4	<35 °C	>35 °C	35.0 °C ± 1.1 °C/35.4 ± 1.0 °C	30 min	NP	2.21	1.24	3.92
Frisch et al. (14)	Cohort	America	A retrospective chart review of Henry Ford Health System, and Wayne State University School of Medicine.	1,525 (549/947)	NP	77.6 ± 14.2	Defined as those who demonstrated a mean intraoperative temperature less than 36 °C.	>36 °C	NP	30 days	6	3.3	1.19	9.14
Brown et al. (16)	Case-control	America	(1) The Perioperative DataMart, and (2) Mayo Clinic Life Science Services and the Data Discovery Query Builder (MCLSS/DDQB). record	5,018 (2,546/2,472)	1,335, 26.6%	60	Temperature below 36 °C	Temperature greater than 36 °C	35.7 ± 0.7/35.7 ± 0.7	30 days	NP	0.98	0.9	1.5
Eng et al. (15)	Cohort	America	A prospectively collected institutional database at City of Hope National Medical Center	170 (53/117)	14, 8.2%	41	Temperature less than 36.0 °C within the 30 min before or the 15 min immediately after anesthesia time	>36 °C	NP	The 30 min before or the 15 min immediately after anesthesia time	NP	1.04	1.01	1.07
Baucom et al. (17)	Cohort	America	Included adult paients who underwent elective segmental colectomy by 1 of 4 board-certified colorectal surgeons at a single institution from January 1, 2005, through December 31, 2009.	295 (153/142)	36, 12.2%	61.8	Temperature of less than 36.0 °C	>36 °C	NP	30 days	NP	1.17	0.76	1.81

### The overall association between preoperative hypothermia and SSI

**Figure 2:**
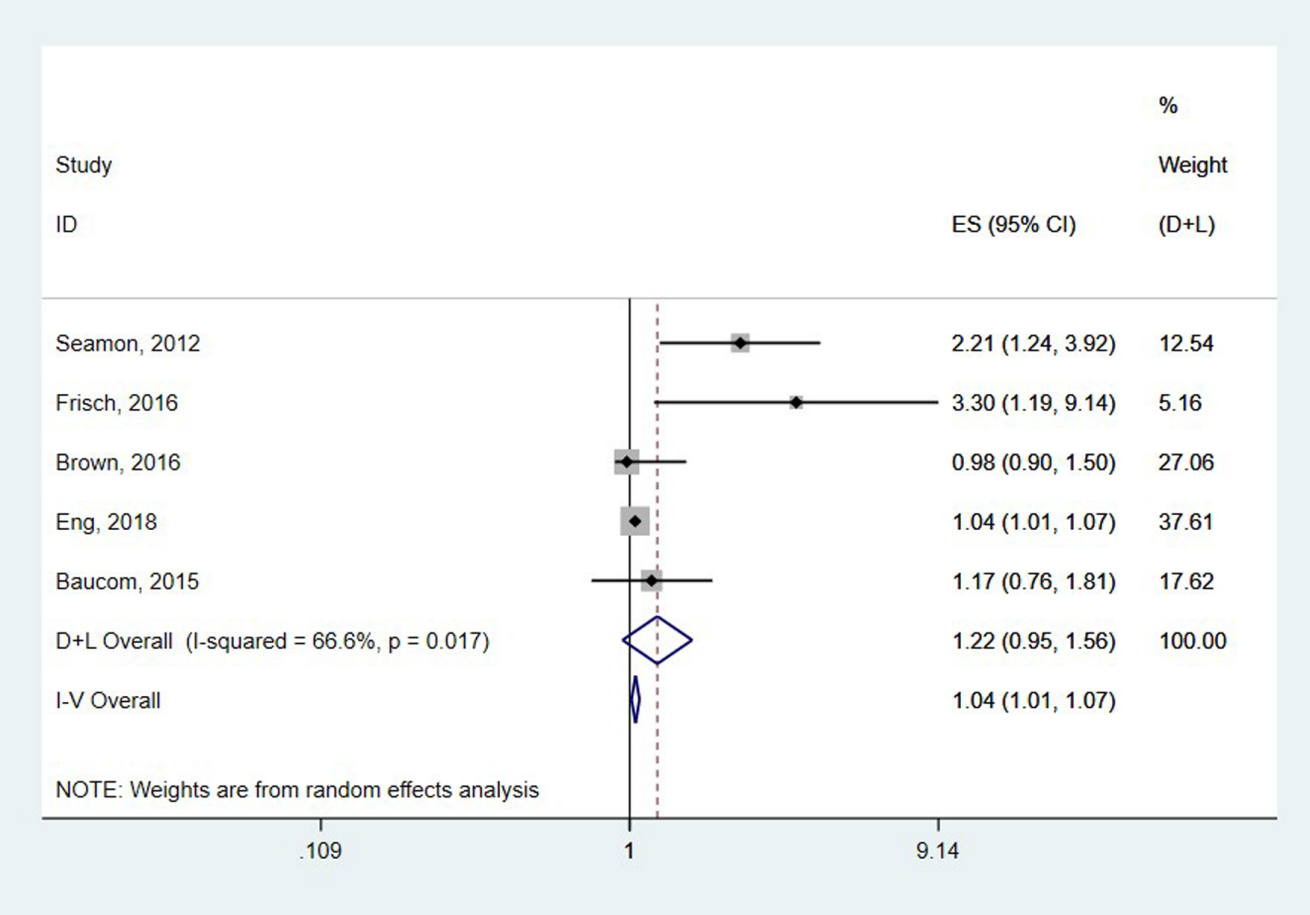
The overall association between Preoperative hypothermia and SSI.

The pooled results from five studies involving 6,002 subjects showed no association between intraoperative hypothermia and SSI (HR = 1.22, 95% CI: 0.95–1.56, *P* = 0.119) and high heterogeneity (I^2^ = 66.6%, *P* = 0.017) (Figure 2).

A stratified analysis was performed to explore the effects of study design and age on this association. A subgroup analysis showed that there was little difference in the association between intraoperative hypothermia and SSI for either study design or generation, with no effect on SSI in patients younger than 50 years and those older than 50 years with intraoperative hypothermia (HR = 1.43, 95% CI: 0.69–2.97, *P* = 0.335 vs. HR = 1.25, 0.80–1.95, *P *= 0.420, respectively). The HR difference between the risk of SSI in patients with intraoperative hypothermia was not statistically significant in either the cohort or case-control studies (Table 3).

**Table 3:** Subgroup analysis of the association between intraoperative hypothermia and risk of surgical site infections.

Subgroup	No. of studies	Hazard ratio	95% CI	P_heterogeneity_	I^2^ static	*P*_overall effect_
Total	4	1.22	0.95–1.56	0.017	66.6	0.119
Age						
<50	2	1.43	0.69–2.97	0.01	84.8	0.335
>50	3	1.25	0.80–1.95	0.071	62.3	0.333
Study design						
Cohort	3	1.46	0.95–2.24	0.008	74.5	0.081
Case-control	1	0.89	0.90–1.50	NA	NA	0.877

### Publication bias

**Figure 3:**
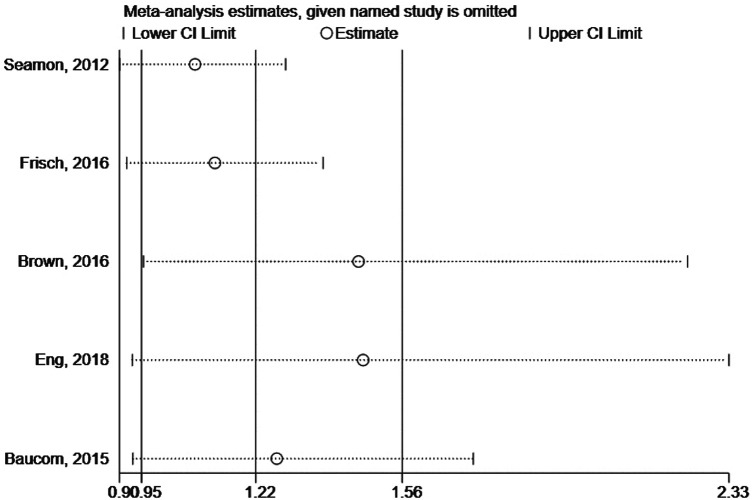
Sensitivity analysis of the SSI risk in patients with Intraoperative hypothermia.

We assessed the impact of individual studies on the overall results by excluding one study at a time and combining the remaining studies. When any study was banned, the combined HR did not significantly change, indicating that the current study overall results were robust (Figure 3).

### Sensitivity analysis

Funnel plots were not constructed because the number of included studies was less than ten. Due to the high degree of heterogeneity, we have reported more conservative results in the Results section using a random-effects model, whereas the pooled results from the fixed-effects model show that the findings remain the same for all groups. The random-effects model incorporates both within-study and between-study variability, allowing for a more accurate estimation of the overall effect size. (Table 4).

**Table 4:** Comparison of the use of random-effects vs. fixed-effects models.

Analysis	I^2^ static	HR (95% CI), Random-effects model	HR (95% CI), Fixed-effects model
Overall	66.6%	1.27 (0.93–1.73)	1.04 (1.01–1.07)
<50	84.8%	1.43 (0.69–2.97)	1.04 (1.01–1.07)
>50	62.3%	1.62 (0.50–5.23)	1.05 (0.82–1.35)
Cohort	74.5%	1.75 (0.85–3.59)	1.04 (1.01–1.07)
Case-control	NA	0.98 (0.90–1.50)	0.98 (0.90–1.50)

## Discussion

### Principal findings

In this meta-analysis, a total of five studies were evaluated to determine the role of intraoperative hypothermia during the development of SSI. The methodological quality was assessed in different ways owing to different study designs. Our results showed no association between intraoperative hypothermia and SSI (HR = 1.22, 95% CI: 0.95–1.56, *P* = 0.134). Notably, this risk was similar for individuals aged >50 years and <50 years. Age did not significantly affect the relative risk of SSI in the patients with intraoperative hypothermia.

### Potential interpretations of the results

Notably, several studies have reported conflicting results regarding the association between hypothermia and SSI. A study by Seamon MJ et al. (13) found that maintaining an intraoperative body temperature above 35 °C is crucial in reducing the incidence of SSI following traumatic laparotomy. Intraoperative hypothermia has also been identified as a risk factor of SSI in patients undergoing elective colorectal surgery (15) and orthopedic procedures. These findings are consistent with those of Frisch et al. regarding hip fracture surgery (14). However, not all studies have shown a link between intraoperative hypothermia and SSI rates. Brown et al. (16) found no correlation across all Class I/clean surgical trauma patients in various specialties. The varying nature of these patient groups and surgical procedures may account for discrepancies in the results. Furthermore, a retrospective cohort study by Baucom et al. (17) that examined the incidence and duration of intraoperative hypothermia during colectomies did not find a significant difference in the SSI rates between normothermic and hypothermic patients. Patients with orthopedic conditions, especially hip fractures, have distinct physiological traits and co-occurring health conditions, and require specialized care. More high-quality research is still needed to clarify this relationship, especially for specific patient groups like orthopedic and trauma patients that have unique clinical characteristics and perioperative care needs. Standardized reporting and control for potential confounders in future research may help generate more definitive conclusions.

A recent study by Wagner et al. explored the relationship between perioperative hypothermia and surgical site infection (SSI) in pediatric patients. Surprisingly, their findings differed from previous studies conducted on adult patients. Wagner et al. found that mild perioperative hypothermia in pediatric patients was associated with a lower incidence of SSI, which contrasts with the findings from adult studies that suggest an increased risk of SSI with perioperative hypothermia. The authors suggest that the aggressive measures taken to prevent perioperative hypothermia in adult patients may not have the same beneficial impact on preventing SSI in pediatric patients. This highlights the importance of considering potential differences between pediatric and adult patients when addressing this clinical issue. However, it is important to note that the number of studies focusing on pediatric patients was limited in our review, and most of the included studies only examined adult patients. Therefore, further high-quality research is needed to investigate the relationship between perioperative hypothermia and SSI specifically in pediatric populations. The findings of Wagner et al. underscore the need for more comprehensive studies in this area.

Although our study did not find a link between intraoperative hypothermia and SSI, we recommend ongoing efforts to prevent perioperative hypothermia. Maintaining normothermia during surgery can improve patient outcomes and reduce mortality rates, ischemic cardiovascular events, blood component therapy requirements, and the length of hospital stay. However, further research is needed to fully understand the relationship between intraoperative hypothermia and SSI. While we were able to provide the mean temperature differences for two studies in Table 2, we recognize that the remaining studies did not report this information. This omission may have implications for the ability to detect an effect if the temperature differences were too low. To address this limitation, we emphasize the need for future studies to report not only the mean temperature differences but also the duration of time spent below specific temperature thresholds, such as “time below 36/35 °C”. This additional information would provide a more comprehensive understanding of the potential impact of intraoperative hypothermia on surgical site infection rates.

By highlighting this need for improved reporting in future studies, we aim to encourage researchers to provide more detailed information on temperature differences and duration, which will enhance the validity and applicability of the findings in this field.

### Comparison with previous meta-analysis

Our systematic review aims to contribute to the ongoing debate by providing an updated synthesis of the evidence on the association between intraoperative hypothermia and surgical site infection (SSI). Contrary to the findings of previous meta-analyses, such as the study by Xu et al. (24), which suggested an increased risk of SSI with hypothermia, our review indicates that the evidence does not support such a strong association. One key difference lies in the study selection criteria. While Xu et al. focused primarily on randomized controlled trials, our review included observational studies. This inclusive approach allowed us to capture a more diverse range of clinical scenarios and patient populations, potentially leading to a more nuanced understanding of the effects of intraoperative hypothermia on SSI risk. Moreover, the definitions and methodologies used to diagnose SSI across studies varied, which could have contributed to the discrepancies in our findings. Consistent criteria for SSI diagnosis are essential for accurate comparison and interpretation of the data.

Another factor that may account for the differences is the quality of the studies included. Our review may have favored studies with higher methodological rigor, which could have influenced the reliability of our results. Additionally, the clinical context and anthropometric factors play a significant role in the risk of SSI. Differences in patient populations, surgical procedures, and healthcare settings could contribute to the varying associations observed between hypothermia and SSI.

Considering these considerations, it is crucial to recognize that the relationship between intraoperative hypothermia and SSI is complex and may be influenced by several interrelated factors. Our systematic review highlights the need for future research to harmonize study designs, outcomes, and definitions to provide clearer and more conclusive evidence.

### Limitations

Our study has several limitations that should be considered when interpreting the results. First, the included studies were all published between 2012 and 2018, which may not reflect the most current advancements in equipment, treatment, and prevention methods. Our retrospective study design did not allow us to determine the causation between the parameters and outcomes. Therefore, more high-quality prospective studies are required to thoroughly investigate this correlation, particularly because hypothermia may not be apparent before surgery. Second, we included a small number of studies, with only five analyzed studies, one of which was a case-control study. This limits our ability to draw firm conclusions regarding the relationship between intraoperative hypothermia and SSI. Third, there were variations in standardized protocols for temperature measurement among the studies analyzed without testing the reliability of temperature measurement tools or measuring the duration of hypothermia and intraoperative temperature. These variations could affect the accuracy of the results. Finally, we excluded studies that did not provide OR or HR, potentially introducing selection bias. Although we considered including studies with other relevant data, we were unable to locate such studies during our research process.

As only two studies reported mean temperature differences, this omission in other studies may impact ability to detect effects if differences were small. We emphasize the need for future research to report not only mean temperature differences but also duration below thresholds like 36/35 °C. Such detailed temperature data would provide comprehensive understanding and strengthen validity/applicability of findings regarding hypothermia's potential impact on SSI rates.

Future studies should aim to provide more detailed information on temperature differences and the duration of time spent below specific temperature thresholds. Doing so will enable researchers to better evaluate the influence of intraoperative hypothermia on SSI risk and potentially identify optimal temperature thresholds for effective prevention strategies.

## Conclusion

In conclusion, we found no association between intraoperative hypothermia and subsequent SSI, although the relationship between hypothermia and SSI in all surgical patients remains unclear. Careful consideration of the multifactorial etiology of SSI in future prospective studies on hypothermia would further enhance the strength of these analyses. In future studies, more randomized controlled trials are needed to determine the role of intraoperative hypothermia in the development of SSI in high-risk surgical procedures and patient populations.

## Data Availability

The original contributions presented in the study are included in the article/Supplementary Material, further inquiries can be directed to the corresponding authors.
